# Identification and response analysis of xyloglucan endotransglycosylase/hydrolases (XTH) family to fluoride and aluminum treatment in *Camellia sinensis*

**DOI:** 10.1186/s12864-021-08056-5

**Published:** 2021-10-25

**Authors:** Zichen Wu, Chuanlei Cui, Anqi Xing, Xiaohan Xu, Yi Sun, Zhiqiang Tian, Xuyan Li, Jiangyuan Zhu, Genmei Wang, Yuhua Wang

**Affiliations:** 1 College of Horticulture, Nanjing Agricultural University, Nanjing, 210095 China; 2 Co-innovation Center for Sustainable Forestry in Southern China, Nanjing Forestry University, Nanjing, 210037 China

**Keywords:** *Camellia sinensis*, Xyloglucan endotransglycosylase/hydrolases (XTH), Aluminum, Fluoride

## Abstract

**Background:**

Xyloglucan endotransglycosylase/hydrolases (XTH) can disrupt and reconnect the xyloglucan chains, modify the cellulose-xyloglucan complex structure in the cell wall to reconstruct the cell wall. Previous studies have reported that XTH plays a key role in the aluminum (Al) tolerance of tea plants (*Camellia sinensis*), which is a typical plant that accumulates Al and fluoride (F), but its role in F resistance has not been reported.

**Results:**

Here, 14 *CsXTH* genes were identified from *C. sinensis* and named as *CsXTH1–14*. The phylogenetic analysis revealed that CsXTH members were divided into 3 subclasses, and conserved motif analysis showed that all these members included catalytic active region. Furthermore, the expressions of all *CsXTH* genes showed tissue-specific and were regulated by Al^3+^ and F^−^ treatments. *CsXTH1*, *CsXTH4*, *CsXTH6*–*8* and *CsXTH11–14* were up-regulated under Al^3+^ treatments; *CsXTH1–10* and *CsXTH12–14* responded to different concentrations of F^−^ treatments. The content of xyloglucan oligosaccharide determined by immunofluorescence labeling increased to the highest level at low concentrations of Al^3+^ or F^−^ treatments (0.4 mM Al^3+^ or 8 mg/L F^−^), accompanying by the activity of XET (Xyloglucan endotransglucosylase) peaked.

**Conclusion:**

In conclusion, CsXTH activities were regulated by Al or F via controlling the expressions of *CsXTH* genes and the content of xyloglucan oligosaccharide in *C. sinensis* roots was affected by Al or F, which might finally influence the elongation of roots and the growth of plants.

**Supplementary Information:**

The online version contains supplementary material available at 10.1186/s12864-021-08056-5.

## Background

The tea plant [*Camellia sinensis* (L.) O. Kuntze] is one of the economic crops in China, which can be processed into an important non-alcoholic beverage [1]. Tea plants can accumulate large amounts of aluminum (Al) and fluoride (F), which are partially dissolved in the tea soup [2]. Excessive Al intake over the long term is easy to induce Alzheimer’s disease and excessive F intake may cause serious health problems [2, 3]. Therefore, the study on the mechanism of absorption and accumulation of Al and F in *C. sinensis* plays a vital role in providing theoretical basis for reducing the content of Al and F and improving the quality of tea.

The mechanism of Al and F accumulation is closely related to the construction of cell walls in *C. sinensis*. As a thick wall existing in the periphery of cells, cell wall is the first to sense environmental stress. In the process of the growth and differentiation of cells, the cell walls must be relaxed and extended before the elongation of roots. Previous studies have shown that Al in *C. sinensis* is mainly accumulated in the cell walls. The more mature the leaf is, the thicker the cell wall is and the higher the content of Al is [4]. The combination of Al with the cell walls of roots can reduce the elasticity and hydraulic conductivity of cell walls, inhibiting root elongation. In addition, studies have shown that enrichment of F in the cell wall can prevent F from entering cells, which is an important mechanism of F tolerance in *C. sinensis* [5, 6].

Xyloglucan endotransglycosyltransferase/hydrolase (XTH) widely exists in various plant tissues, and catalyzes the cleavage and polymerization of xyloglucan molecules, thereby remodeling the cell wall cellulose-xyloglucan complex structure, enabling cell wall remodeling [7]. This process can minimize the resistance and promote the elongation of cells to help cell walls loosen and expand. In addition, XTH can also catalyze the transfer of newly synthesized xyloglucan molecules to the original cell wall network structure in order to maintain the thickness and integrity of the cell wall during reconstitution [8]. XTHs belongs to glycoside hydrolase family (GH16), and the most suitable substrate is xyloglucan, which mainly exerts two activities: one is xyloglucan endotransglucosylase (XET) activity, which catalyzes the cleavage of the p-1, 4-glycosidic bond in the xyloglucan molecule and transfers the resulting glycosyl terminus to the non-reducing end of another xyloglucan or oligosaccharide. And the other is xyloglucan hydrolase (XEH) activity, which catalyzes the hydrolysis of xyloglucan [8–11]. XTHs are a class of proteins encoded by multiple genes that were originally divided into three subfamilies, I, II and III. Yokoyama et al. (2004) compared the XTH sequences in the whole genome of *Oryza sativa* and *Arabidopsis thaliana* and found no significant difference between subfamilies I and II, so they were combined and named as I/II [12]. Baumann et al. (2007) also classified subfamilies III into two subgroups, IIIA and IIIB, respectively, based on their catalytic activity [13]. Previous studies have shown that members of subfamilies I, II, and IIIB exert XET activity primarily [14–17].

In recent years, the response of *XTHs* to stress has become the focus of researchers’ attention because *XTHs* are a class of genes that regulate cell elongation and expansion during the growth of plants [18, 19]. For example, 3 homologous genes, *CaXTH1*, *CaXTH2* and *CaXTH3*, were found in *Capsicum annuum* to cope with stress such as drought, high salt and low temperature [20]. Yang et al. (2011) reported that heavy metal Al^3+^ stress significantly down-regulated the transcriptional expression of *AtXTH14*, *AtXTH15* and *AtXTH31* in *A. thaliana*, which would reduce the activity of XET, resulting in a significant inhibition of root elongation [21]. As a result of the important role of cell walls playing in the process of Al and F accumulation and resistance in plants, the function of *XTHs* is also of great significance in the study of the molecular mechanism of this process. To reveal the effects of *CsXTHs* on cell wall reconstruction under Al and F treatments in *C. sinensis*, we analyzed the bioinformatics characteristics of *CsXTH*s, measured the expression levels of *CsXTH*s, the content of xyloglucan and xyloglucan oligosaccharide, and determined XET activities under Al and F treatments in the present study.

## Results

### Identification and phylogenetic tree analysis of *XTHs* in *C. sinensis*

Based on the transcriptomic databases of *C. sinensis*, 45 *XTHs* sequences were screened [22]. After further verification by BLAST program and ClustalX l.83 software, 14 *CsXTH* genes were identified and renamed as *CsXTH1* to *CsXTH14.*

**Fig. 1:**
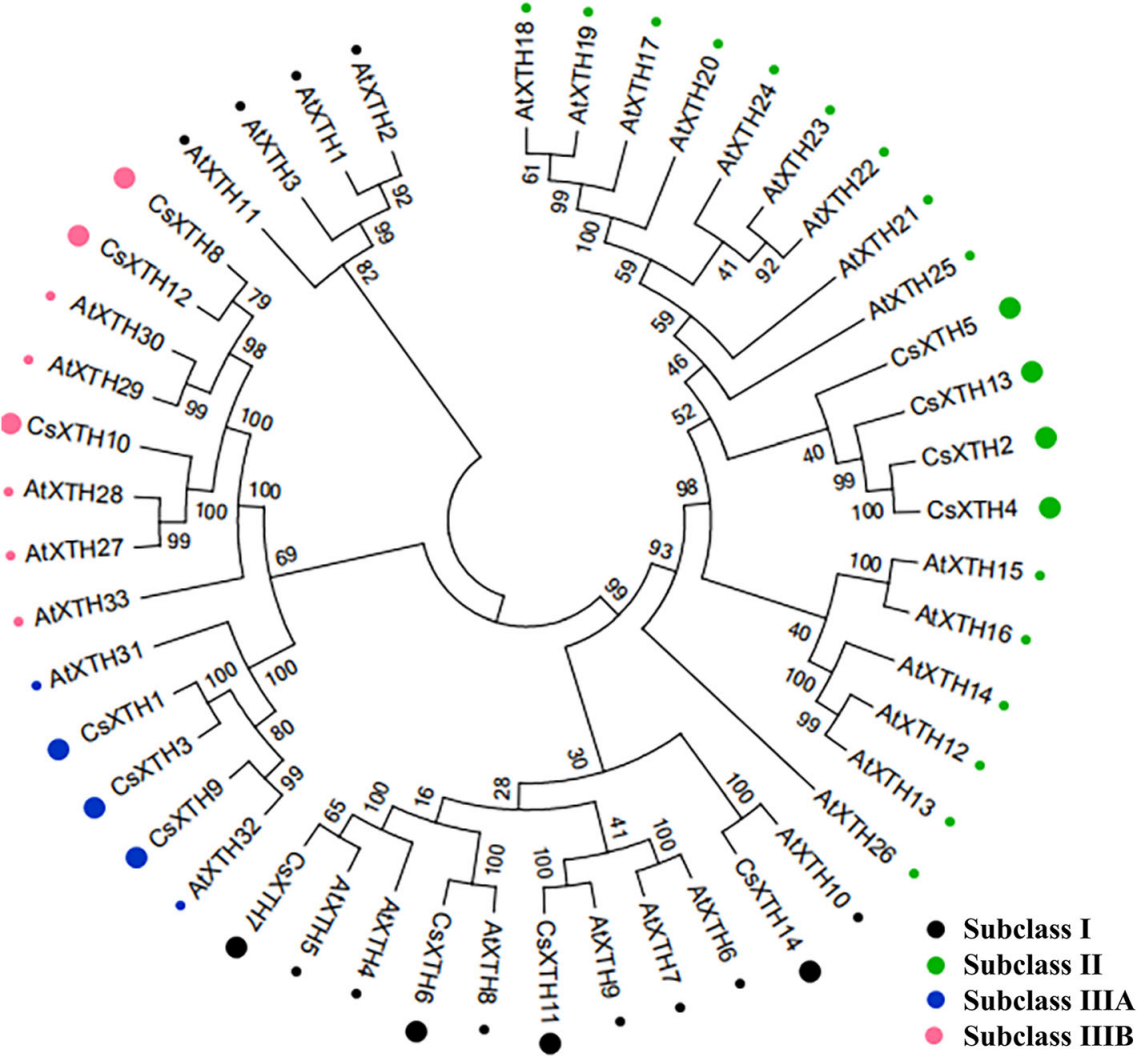
The phylogenetic tree of XTHs members from *Camellia sinensis* and *Arabidopsis thaliana*. Different subclasses are marked with dots of different colors (black dots: subclass I; green dots: subclass II; blue dots: subclass IIIA; pink dots: subclass IIIB)

The phylogenetic tree was constructed with 14 CsXTHs proteins in *C. sinensis* and 33 AtXTHs proteins in *A. thaliana*. All XTHs proteins were divided into three subclasses (subclasses I, II, and III). There were 4 CsXTH members (CsXTH6, CsXTH7, CsXTH11 and CsXTH14) and 11 AtXTH members (AtXTH1–11) belonging to subclass I. And 4 CsXTH members (CsXTH2, CsXTH4, CsXTH5 and CsXTH13) and 15 AtXTH members (AtXTH12–26) belonged to subclass II. Subclass III could be divided into IIIA and IIIB, of which subclass IIIA contained 3 CsXTH members (CsXTH1, CsXTH3 and CsXTH9) and 2 AtXTH members (AtXTH31–32), subclass IIIB contained 3 CsXTH members (CsXTH8, CsXTH10 and CsXTH12) and 5 AtXTH members (AtXTH27–30 and AtXTH33) (Fig. 1).

The physicochemical characteristics of 14 CsXTH proteins were further analyzed. The number of amino acids (aa) of CsXTH proteins varied from 287 (CsXTH13) to 477 (CsXTH12), and their molecular weights ranged from 32,262.41 to 54,695.61 (Table 1). The theoretical isoelectric points of CsXTHs varied from 4.82 (CsXTH6) to 9.92 (CsXTH12) (Table 1). The grand averages of hydropathicity (GRAVY) of CsXTHs were negative, so all CsXTH proteins were hydrophilic. The results of instability index of 14 CsXTHs showed that CsXTH proteins from subclass I were all stable and CsXTH proteins from subclass III (IIIA and IIIB) were all unstable. Half of members (CsXTH2 and CsXTH4) of subclass II were unstable, and the others (CsXTH5 and CsXTH13) were stable.

**Table 1:** Physicochemical characteristics of XTHs in *C. sinensis*

Protein Name	Number of amino acids	Molecular weight	Theoretical pI	Instability index	Grand average of hydropathicity (GRAVY)
CsXTH1	291	32,869.60	5.33	49.63	−0.469
CsXTH2	289	32,401.32	5.57	44.11	−0.313
CsXTH3	291	32,831.56	5.95	45.29	−0.529
CsXTH4	290	32,553.36	5.38	44.71	−0.363
CsXTH5	333	37,996.93	8.74	32.97	−0.498
CsXTH6	299	34,894.80	4.82	34.44	−0.543
CsXTH7	295	34,061.39	7.65	33.85	−0.467
CsXTH8	341	39,371.45	8.88	40.92	−0.560
CsXTH9	295	34,104.87	9.47	53.64	−0.393
CsXTH10	329	37,275.84	6.31	42.98	−0.500
CsXTH11	297	33,850.12	5.87	37.70	−0.301
CsXTH12	477	54,695.61	9.92	54.08	−0.532
CsXTH13	287	32,262.41	8.18	36.45	−0.310
CsXTH14	298	34,590.05	6.08	30.39	−0.403

### Gene structure, conserved motifs and sequence alignment analysis of CsXTHs

**Fig. 2:**
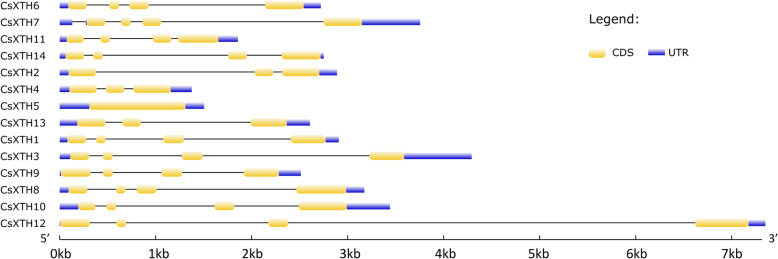
Gene structures of *CsXTH* genes. The yellow rounded rectangles represent coding sequences (CDS); purple rectangles represent untranslated regions (UTR)

The gene structures of 14 *CsXTHs* showed that *CsXTH* genes from subclass I and subclass III (IIIA and IIIB) all had 4 exons (Fig. 2). *CsXTH5* had only 1 exon and other genes of subclass II (*CsXTH2*, *CsXTH4* and *CsXTH13*) all had 3 exons (Fig. 2).

**Fig. 3:**
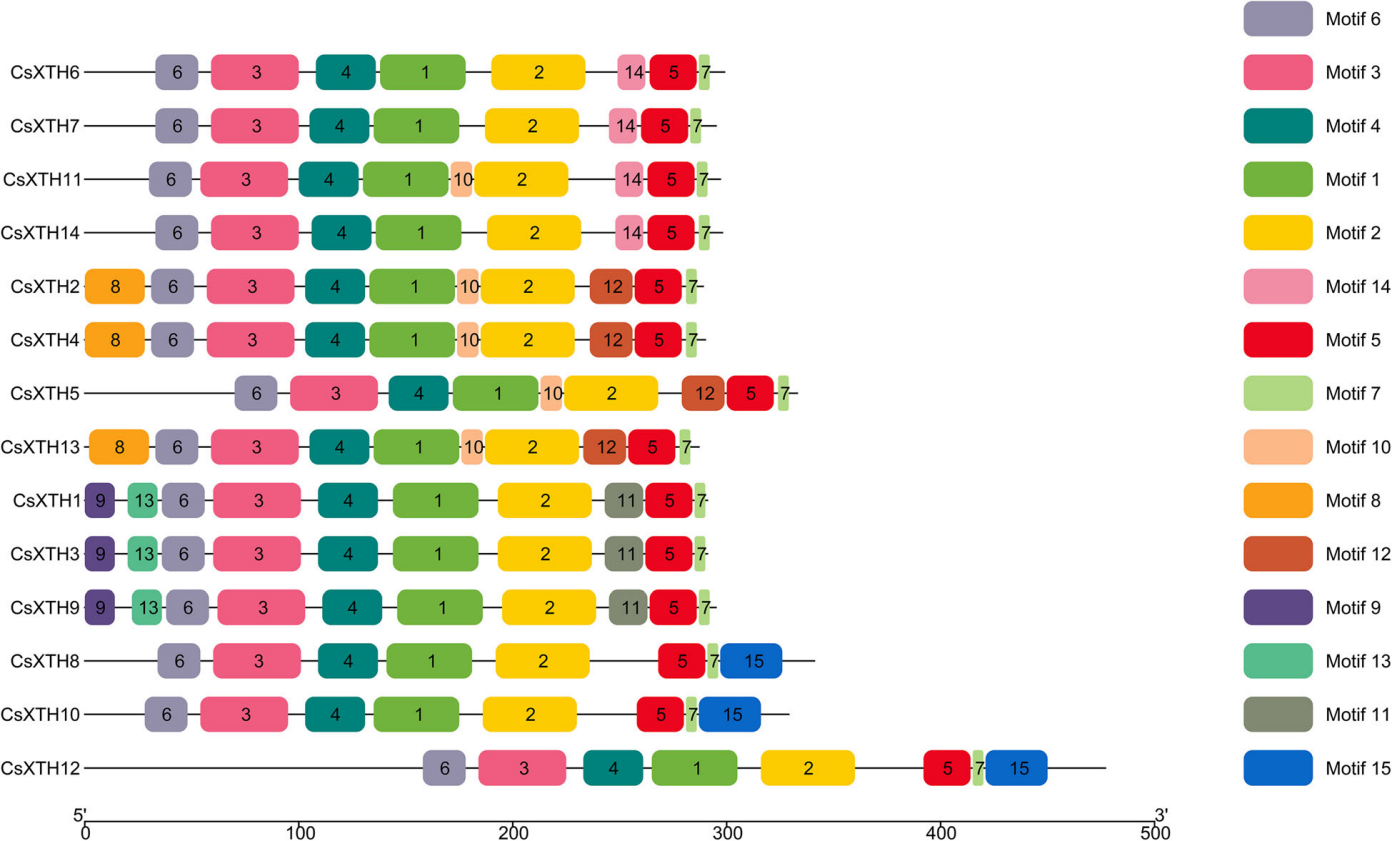
The conserved motifs of CsXTH proteins. Different motifs are represented by color numbered boxes

**Fig. 4:**
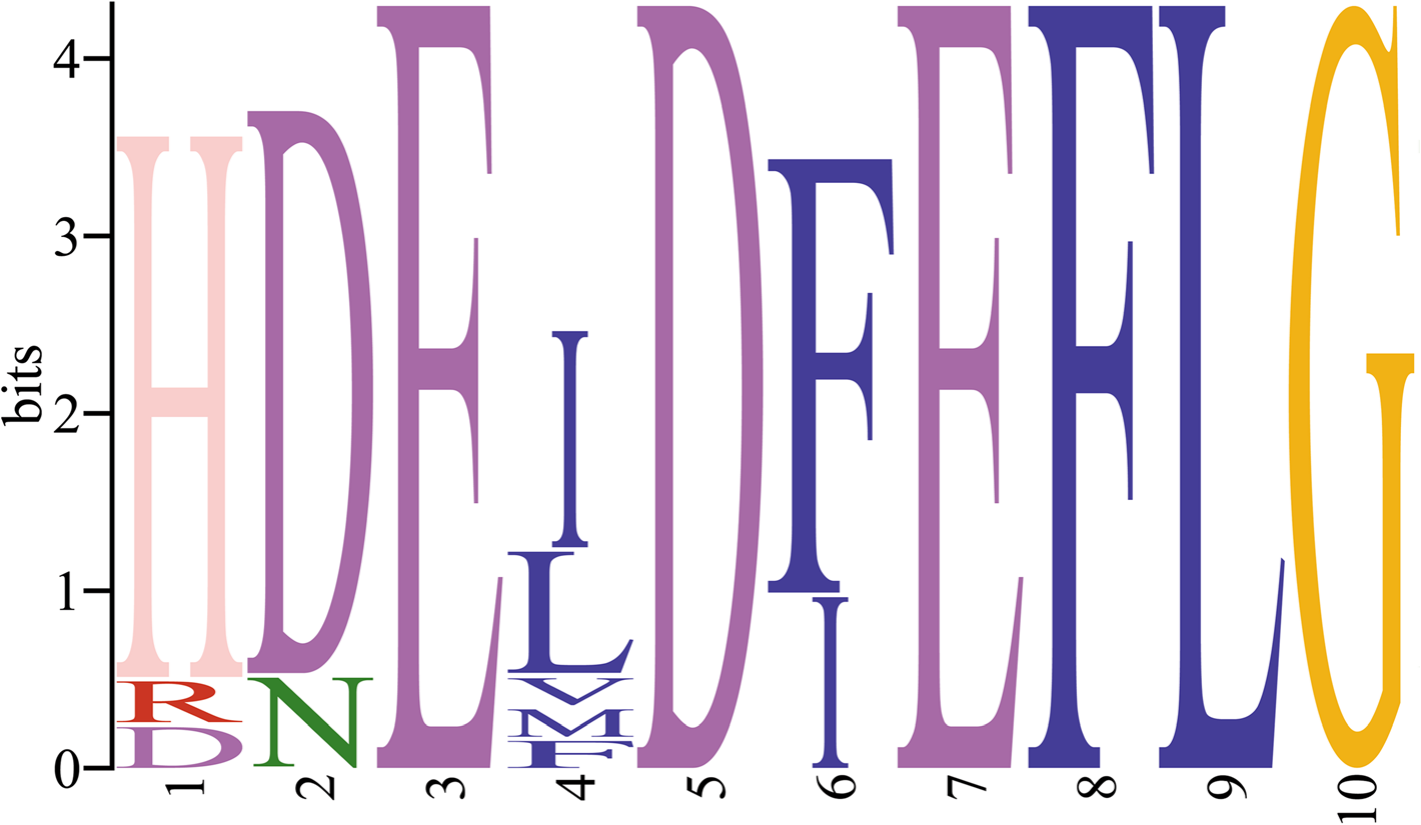
The logo of catalytic sequence motif (HDEIDFEFLG) of CsXTH proteins. The sequence identity of each binding is composed of the superposition of marker (amino acid residues). The height of the superposition reflects the conservatism of the sequence and the height of each marker reflects the frequency that amino acid residue appears at this position

There were 7 conserved motifs (motif 1–7) in all 14 CsXTHs. The conserved motif 4 contained the catalytic sequence motif of the XTH proteins (HDEIDFEFLG) (Fig. 3). And the logo of catalytic sequence motif (HDEIDFEFLG) of CsXTHs was shown in Fig. 4 [23]. Motif 14 was exclusively present in CsXTHs of subclass I, and motif 12 was only existed in CsXTHs of subclass II (Fig. 3). In addition, motif 9, 11 and 13 were only present in CsXTH proteins of subclass IIIA, and motif 15 was exclusively existed in CsXTH proteins of subclass IIIB (Fig. 3).

**Fig. 5:**
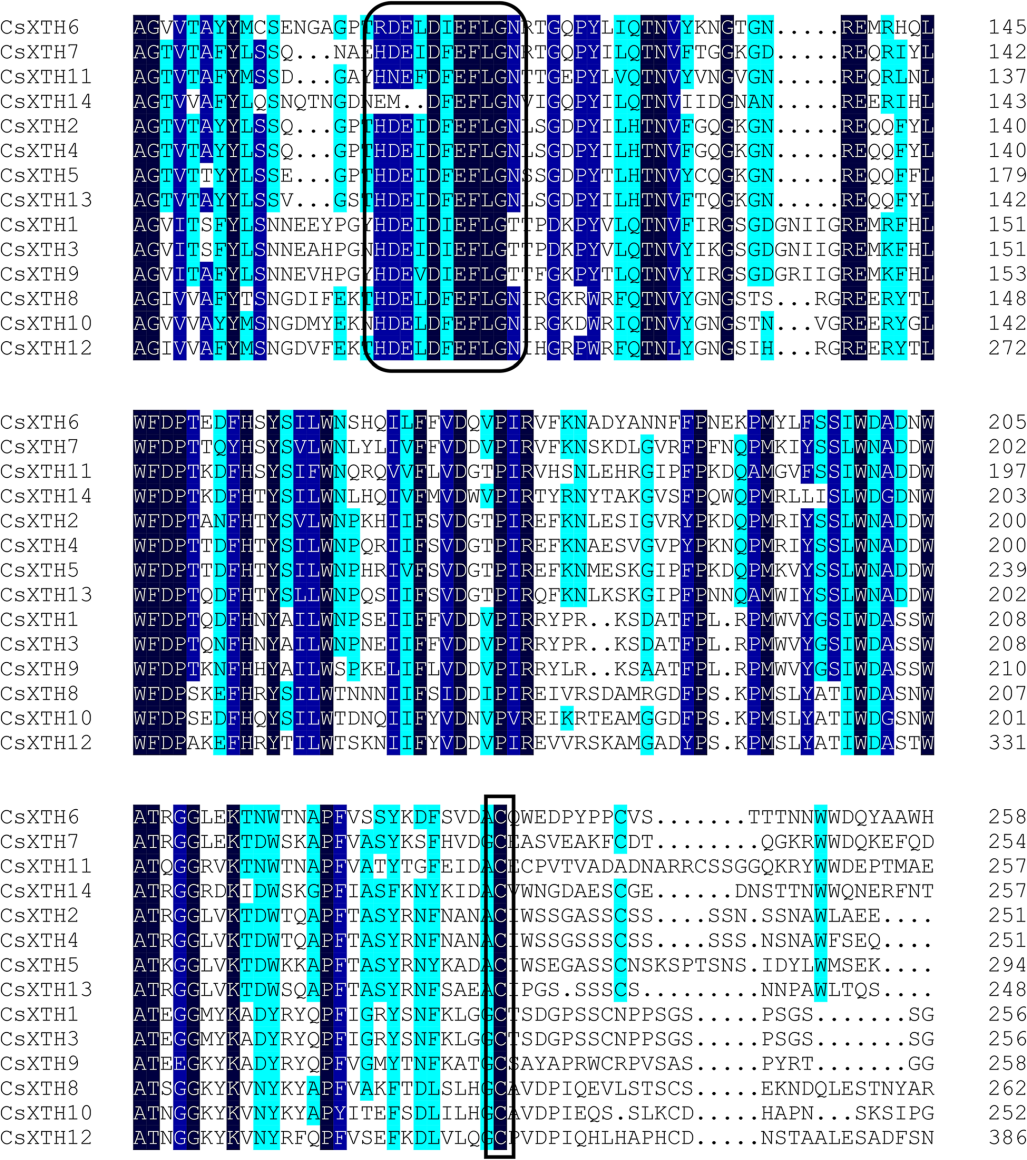
Multiple sequence alignments of 14 CsXTH proteins. The black rounded rectangular frames represent the conserved sequence (HDEIDFEFLG); black rectangular frames represent Cys residues

The multiple sequence alignments of 14 CsXTH proteins indicated that all CsXTH proteins contained the conserved sequence (HDEIDFEFLG) (black rounded rectangular frames) except there were deviations of 1 or 2 amino acids in few sequences (Fig. 5) [23]. Except for all CsXTHs from subclass IIIA (CsXTH1, CsXTH3 and CsXTH9), the catalytic active regions (HDEIDFEFLG) of CsXTH proteins were followed by amino acids of asparagine (N), which were the N-linked glycosylation sites (Fig. 5) [24]. Moreover, conserved Cys residues were present in C-terminal of sequences of all 14 CsXTH proteins (black rectangular frames) (Fig. 5).

### The relative expression levels of *CsXTHs* in different tissues

**Fig. 6:**
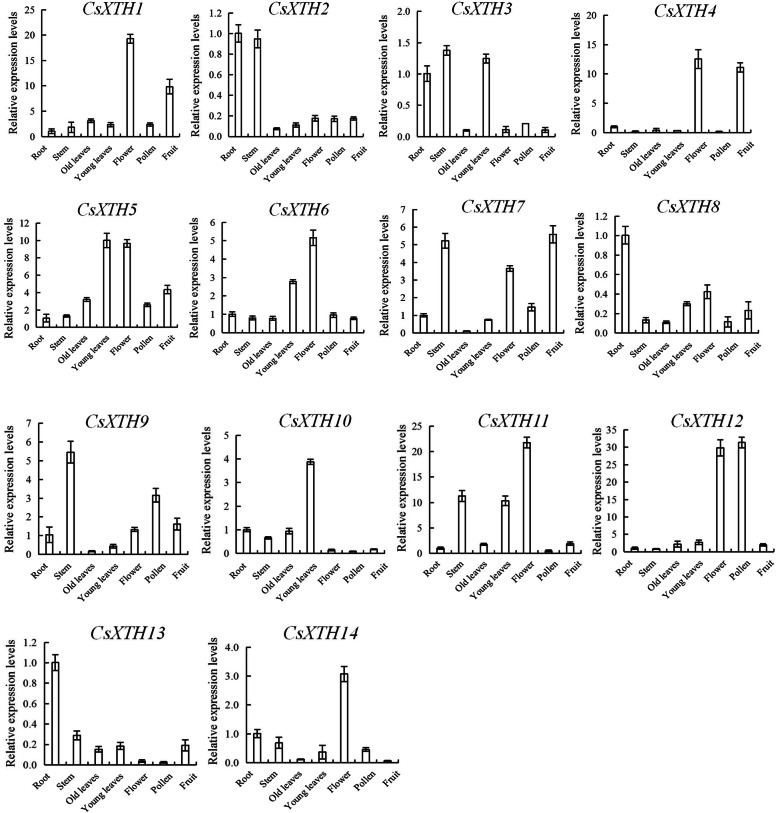
The relative expression levels of 14 *CsXTH* genes in different tissues. Different tissues include root, stem, old leaves, young leaves, flower, pollen and fruit. Each value is the mean of 3 replicates and vertical bars are standard errors (Duncan’s multiple range test; *p* < 0.05)

The relative expression levels of 14 *CsXTH* genes in different tissues (root, stem, old leaf, young leaf, flower, pollen and fruit) of *C. sinensis* cv. Longjing 43 were analyzed by qRT-PCR. The results showed that the expression of 14 *CsXTH* genes in different tissues of *C. sinensis* was significantly different, but the expression level showed certain regularity. *CsXTH2*, *CsXTH3*, *CsXTH8* and *CsXTH13* were highly expressed in roots of *C. sinensis*. The expression levels of *CsXTH2*, *CsXTH3*, *CsXTH7*, *CsXTH9* and *CsXTH11* in stems were relatively high. *CsXTH3*, *CsXTH6*, *CsXTH10* and *CsXTH11* had higher expression level in young leaves of *C. sinensis*. *CsXTH1*, *CsXTH4*, *CsXTH5*, *CsXTH6*, *CsXTH7*, *CsXTH11*, *CsXTH12* and *CsXTH14* were highly expressed in flowers. The expression level of *CsXTH12* in pollens was higher than other genes. In fruits of *C. sinensis*, *CsXTH1*, *CsXTH4*, *CsXTH5* and *CsXTH7* were highly expressed. It was noteworthy that 14 *CsXTH* genes all showed low expression levels in old leaves (Fig. 6). This result indicated that the expressions of *CsXTHs* are tissue-specific.

### The relative expression levels of *CsXTHs* under Al^3+^ treatment

**Fig. 7:**
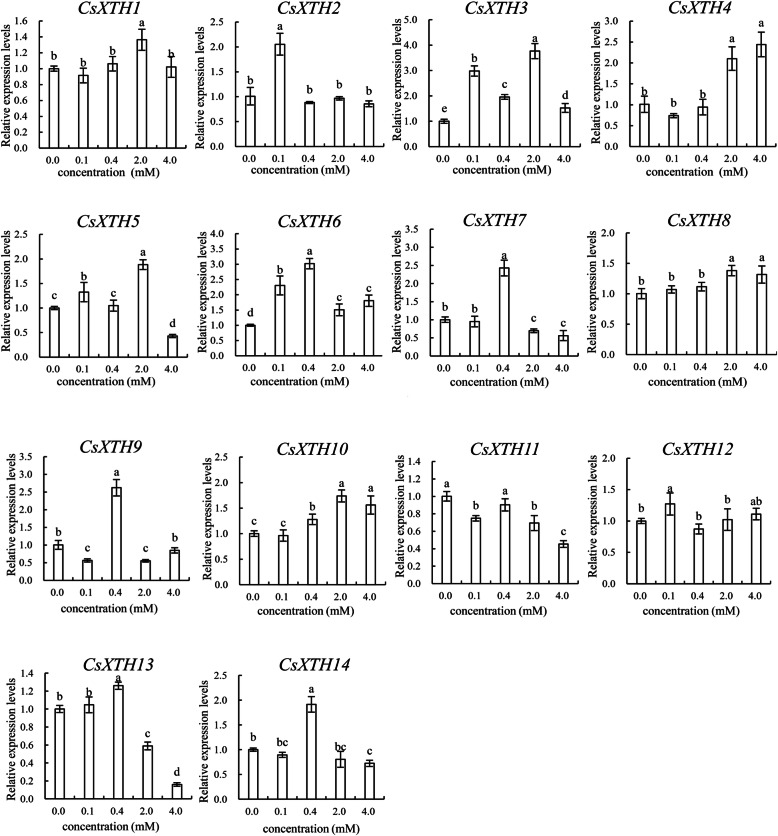
The relative expression levels of 14 *CsXTH* genes under Al^3+^ treatment. The concentrations of Al^3+^ treatment include 0, 0.1, 0.4, 2.0 and 4.0 mM. Each value is the mean of 3 replicates and vertical bars are standard errors. Different lowercase letters indicate significant differences between concentrations (Duncan’s multiple range test; *p* < 0.05)

The *CsXTH* family showed different expression profiles in response to Al^3+^ treatments of different concentrations. The expressions of *CsXTH2*, *CsXTH3*, *CsXTH5*, *CsXTH6* and *CsXTH12* were upregulated under 0.1 mM Al^3+^ treatment. The expression levels of *CsXTH7*, *CsXTH9*, *CsXTH13* and *CsXTH14* significantly increased under 0.4 mM Al^3+^ treatment. The expressions of *CsXTH1*, *CsXTH3*, *CsXTH5* and *CsXTH8* were upregulated under 2.0 mM Al^3+^ and then declined at 4 mM Al^3+^ treatment. The expressions of *CsXTH5*, *CsXTH11* and *CsXTH13* were downregulated under 4.0 mM Al^3+^ treatment, while *CsXTH4* and *CsXTH10* were upregulated under the treatment of 4.0 mM Al^3+^ (Fig. 7).

### The relative expression levels of *CsXTHs* under F^−^ treatment

**Fig. 8:**
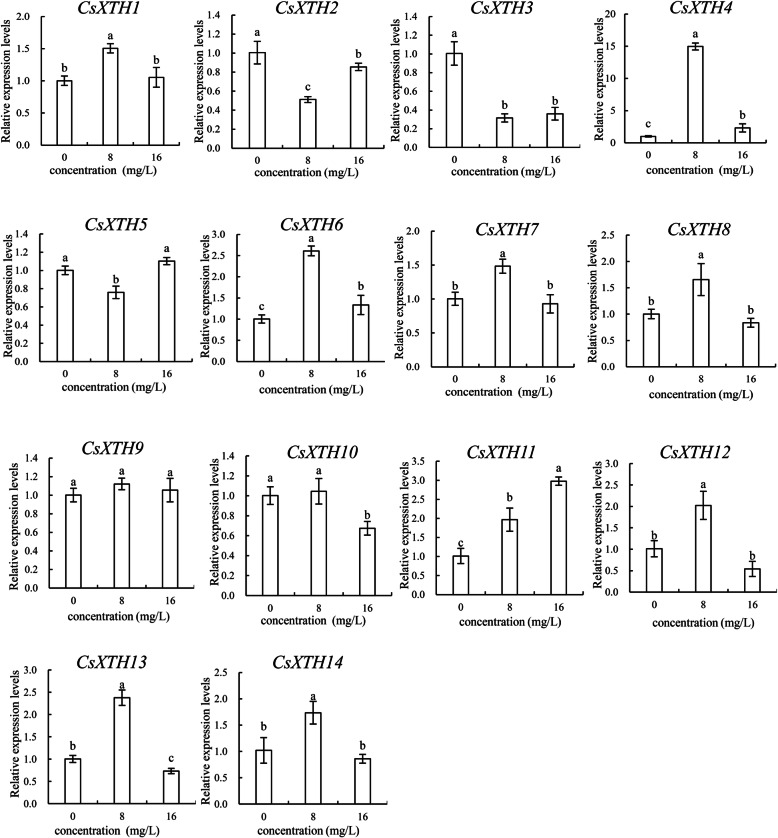
The relative expression levels of 14 *CsXTH* genes under F^−^ treatment. The concentrations of F^−^ treatment include 0, 8 and 16 mg/L. Each value is the mean of 3 replicates and vertical bars are standard errors. Different lowercase letters indicate significant differences between concentrations (Duncan’s multiple range test; *p* < 0.05)

In the present study, the relative expression levels of 14 *CsXTH* genes in roots under 0 mg/L, 8 mg/L and 16 mg/L F^−^ treatment were analyzed, and the results were shown in Fig. 8. The expressions of *CsXTH1*, *CsXTH4*, *CsXTH6*, *CsXTH7*, *CsXTH8*, *CsXTH12*, *CsXTH13* and *CsXTH14* increased to the maximum levels at 8 mg/L F^−^ treatment. However, the expressions of *CsXTH2* and *CsXTH5* decreased at 8 mg/L F^−^ and then slightly increased at 16 mg/L F^−^. Furthermore, *CsXTH2*, *CsXTH5* and *CsXTH11* all showed higher expression levels at 16 mg/L F^−^ treatment compared to that under 8 mg/L F^−^ treatment. Compared with the control (0 mg/L F^−^), expression of *CsXTH3* decreased under 8 mg/L and 16 mg/L F^−^ treatments. It was notably that there was no significant difference in expressions of *CsXTH9* under 8 mg/L and 16 mg/L F^−^ treatments (Fig. 8).

### The immunofluorescence labeling in XTHs and activity of XET

**Fig. 9:**
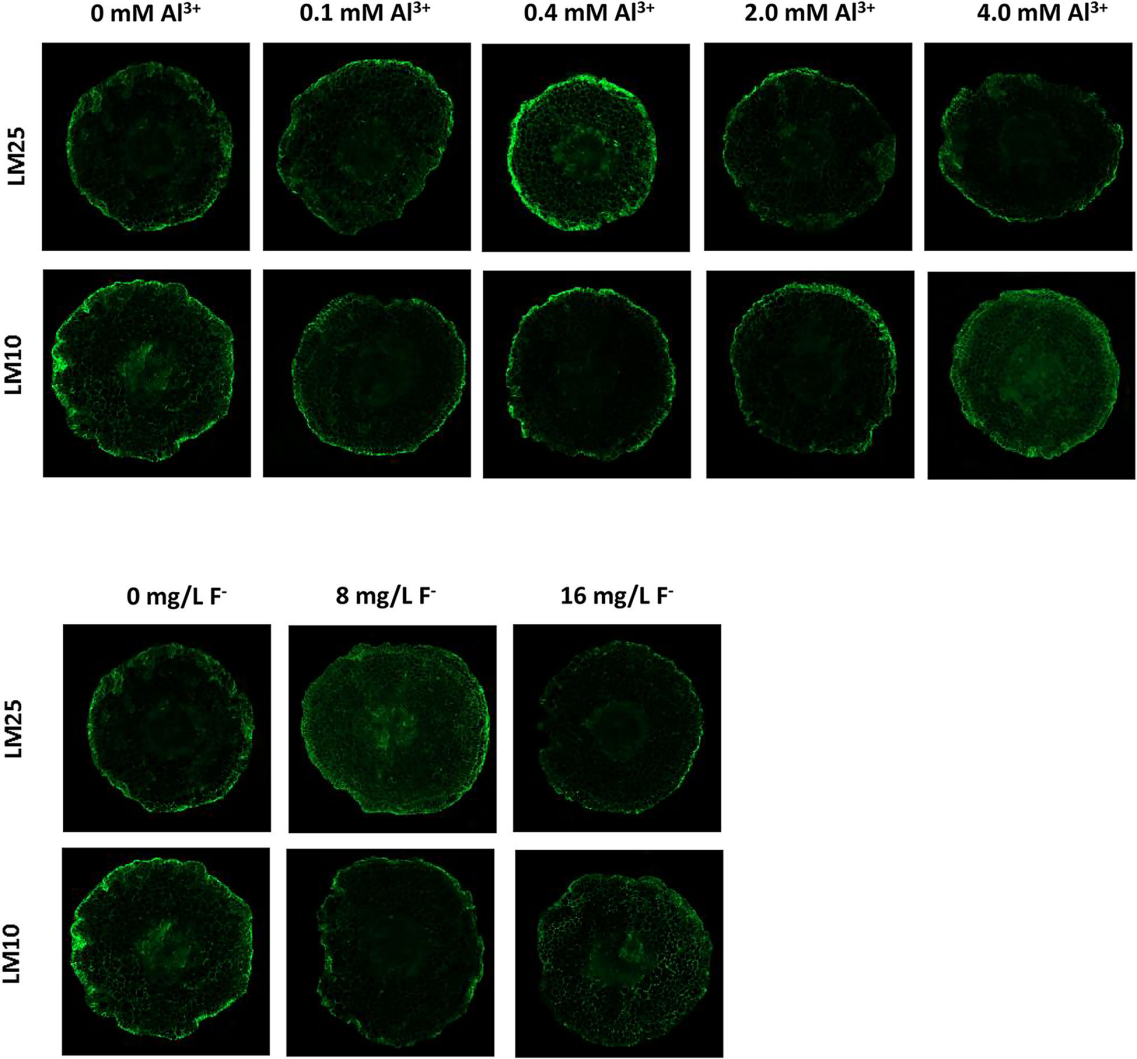
Immunofluorescence localizations of LM10 and LM25 in roots of *C. sinensis* under Al^3+^ and F^−^ treatment. The concentrations of Al^3+^ treatment include 0, 0.1, 0.4, 2.0 and 4.0 mM. The concentrations of F^−^ treatment include 0, 8 and 16 mg/L. The fluorescence brightness of LM10 or LM25 represents the content of xyloglucan or xyloglucan oligosaccharide

The results of immunofluorescence localization of LM10 and LM25 in roots showed that the fluorescence intensity of LM25 was relatively weak but the LM10 was strong without Al^3+^ or F^−^ treatment. With the increase of Al^3+^ concentration, the fluorescence intensity of LM25 grew gradually but LM10 declined. The fluorescence intensity of LM25 peaked under 0.4 mM Al^3+^ and then decreased at 2.0 mM and 4.0 mM Al^3+^ treatments. It was indicated that 0.4 mM Al^3+^ treatment might have a positive effect on the formation xyloglucan oligosaccharide by promoting XTH activity, and higher concentrations of Al^3+^, especially 4.0 mM Al^3+^, could probably inhibit XTH activity. Likewise, treatment of 8 mg/L F^−^ also might promote XTH activity (Fig. 9).

**Fig. 10:**
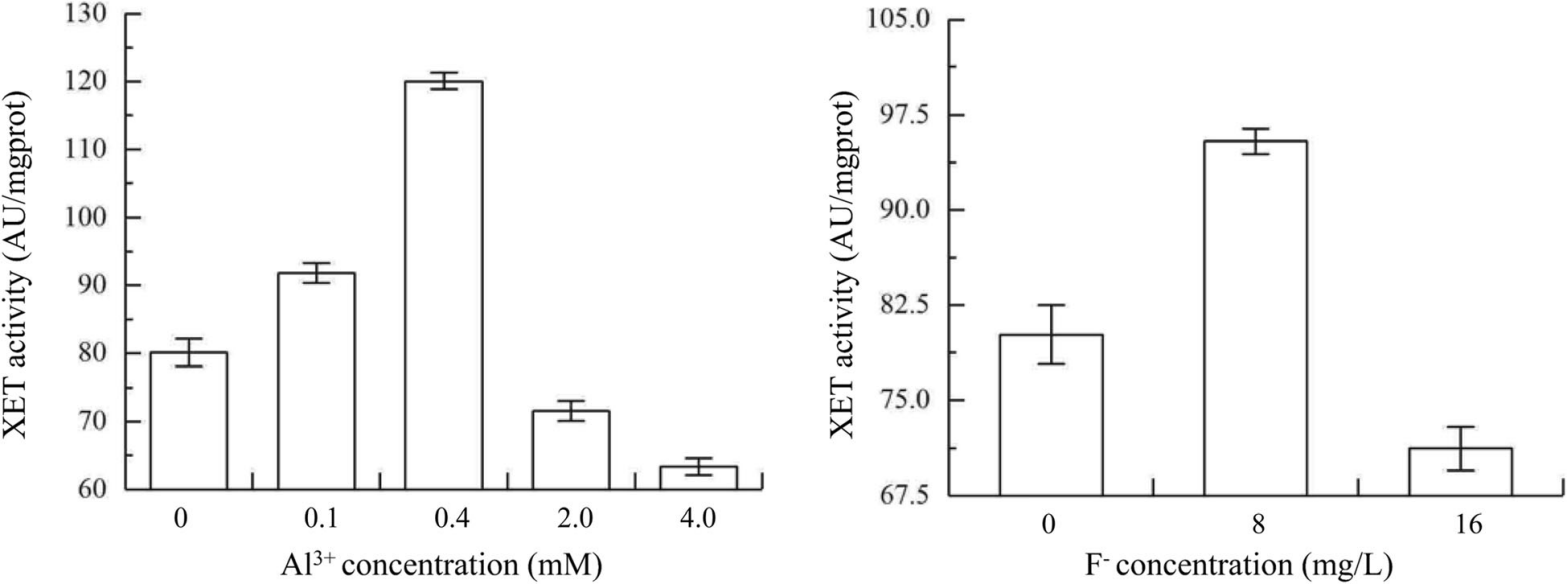
XET activities in roots of *C. sinensis* under Al^3+^ and F^−^ treatment. The concentrations of Al^3+^ treatment include 0, 0.1, 0.4, 2.0 and 4.0 mM. The concentrations of F^−^ treatment include 0, 8 and 16 mg/L. Each value is the mean of 3 replicates and vertical bars are standard errors (Duncan’s multiple range test; *p* < 0.05)

To confirm the results of immunofluorescence labeling, we determined the XET activity, which could catalyse the transformation of xyloglucan. The results showed that activity of XET in roots gradually increased and peaked at 0.4 mM Al^3+^, then decreased under Al^3+^ treatments with higher concentrations (2.0 mM and 4.0 mM) (Fig. 10). Similarly, the XET activity significantly increased under 8 mg/L F^−^, and then decreased with the increase of F^−^ concentration (Fig. 10).

## Discussion

We identified 14 *CsXTH* genes in *C. sinensis* [22]. *CsXTHs* were divided into 3 subclasses: I, II and III as other plants (Fig. 1) [12, 13, 25, 26]. Based on the substrate specificities, XTH family belonged to GH16 [27]. XTHs mainly exerts 2 kinds of catalytic activities: one is XET activity, and the other is XEH activity [28]. Previous studies showed that XET had hydrolytic activity as XEH although XET mainly performed transglucosylase activity [24]. The physicochemical characteristics of CsXTH proteins also illustrated their hydrophilic properties in common (Table 1). Baumann et al. (2007) classified subfamilies III into two subgroups, IIIA (only perform hydrolase activity) and IIIB, respectively, based on their different catalytic activities. The motif analysis showed that all CsXTH proteins contained the catalytic sequence motif (HDEIDFEFLG), which was highly conserved and maintained the activity of XTH, except that there were deviations of 1 or 2 amino acids in some sequences (Fig. 3) [13]. This kind of deviation was also found in XTH family from *A. thaliana* and other species. For example, in *Gerbera jamesonii Bolus*, the sequence motif became HDELDFEFLG since the active site of isoleucine was replaced by leucine [29]. The results of sequence alignment indicated that each CsXTHs had an N-linked glycosylation site following the catalytic active region except for CsXTHs from subclass IIIA, which suggested that N-linked glycosylation site was an essential structure for XTH to perform XET activity (Fig. 5) [30–32]. Therefore, members of subclass IIIA exhibited only hydrolase activity, whereas the reasons for this phenomenon were still unclear [13, 33]. Moreover, conserved Cys residues were present in C-terminal of sequences of all CsXTH proteins (Fig. 5). Studies had shown that these Cys residues could form disulfide bonds and play an important role in the stability of protein structure [34]. The complexity of conserved motifs indicated that CsXTH proteins of *C. sinensis* might have multiple biological functions.

The expression levels of 14 *CsXTHs* genes in different tissues (root, stem, old leaf, young leaf, flower, pollen and fruit) were significantly different (Fig. 6). *CsXTH2*、*CsXTH4*、*CsXTH5* and *CsXTH13* belonged to the same subclass II as *AtXTH17–25* in phylogenetic trees. Studies had shown that *AtXTH18*, *AtXTH19* and *AtXTH20* could promote the elongation of hypocotyl of *A. thaliana*, and *AtXTH21* played an important role in the growth of primary roots [35, 36]. Indeed, *CsXTH2* and *CsXTH13* were highly expressed in roots, which was predicted that *CsXTH2* and *CsXTH13* were functioning in roots [37, 38]. The expressions of *CsXTH4* and *CsXTH5* in flowers were at high levels, which was similar to the expression of *AtXTH21* in flowers [18]. Furthermore, *XTHs* were closely related to plants growth, so it was reasonable that all *CsXTH* genes showed low expression levels in old leaves (Fig. 6).

Some studies showed that Al and F played a key role in growth and development of *C. sinensis*, which was a hyper-accumulator of Al and F [39–41]. The growth and development of *C. sinensis* would be significantly promoted under the treatment of Al at a low concentration (0.29–1.19 mM) [42, 43]. There was a serious inhibition with too high concentrations (> 1.85 mM) of Al [4, 44, 45]. Similarly, there was also a severe inhibition with high concentrations (> 10 mg/L) of F for the growth and metabolism of *C. sinensis* [46, 47].

The expression patterns of *CsXTHs* under Al^3+^ and F^−^ treatments were analyzed to investigate the role of *CsXTH* genes in the process of resisting Al and F. The results showed that the expression patterns of *CsXTHs* in the same subclass were similar under Al^3+^ treatments. For example, 75.0% of *CsXTHs* in subclass I and subclass II and 66.7% of *CsXTH* genes in subclass IIIA were all upregulated under low concentrations of Al^3+^ treatments (0.1 mM, 0.4 mM). This phenomenon might be explained that genes with close genetic relationship had similar functions, which also existed in *XTH* genes of the same subclass between *C. sinensis* and *A. thaliana*. According to the phylogenetic tree, *CsXTH7* and *AtXTH5* had a close relative and belonged to the subclass I. The identity of the protein sequences between CsXTH7 and AtXTH5 was as high as 80.7%. The expression level of *CsXTH7* reached a peak at 0.4 mM Al^3+^ (Fig. 7). Studies showed that the expression of *AtXTH5* was also significantly upregulated under Al stress [31]. The expression levels of *CsXTH*5 and *CsXTH13* decreased when Al^3+^ concentration increased to 4 mM (Fig. 7). Similarly, Yang et al. (2011) reported that the expression levels of *AtXTH14* and *AtXTH15* were dramatically decreased under the Al^3+^ stress, resulting in a reduction of XET activity and a significant inhibition of root elongation in *A. thaliana*, which belonged to subclass II as same as *CsXTH*5 and *CsXTH13* [21]*.* Therefore, we speculated that the down-regulation of the expressions of *CsXTH*5 and *CsXTH13* could be involved in the inhibition process of XTH activity under a high concentration of Al^3+^. In addition, it should be noted that the expression levels of *CsXTH4* and *CsXTH10* increased under 4.0 mM Al^3+^ treatment, which might be related to the response to Al stress in *C. sinensis*.

*CsXTH* also positively responded to F^−^ treatment. The expressions of *CsXTH1*, *CsXTH4*, *CsXTH6*, *CsXTH7*, *CsXTH8*, *CsXTH12*, *CsXTH13* and *CsXTH14* reached the highest levels at 8 mg/L F^−^ (Fig. 8). However, the expression levels of *CsXTH3* all decreased at different concentrations of F^−^, which indicated that F^−^ treatment significantly inhibited the expression of *CsXTH3*. It should be noted that there was no difference in the expression levels of *CsXTH9* at different concentrations of F^−^, indicating that *CsXTH9* was not sensitive to F^−^ treatment. Also, *CsXTH2*, *CsXTH5* and *CsXTH11* all showed higher expression levels at 16 mg/L F^−^ relative to that at 8 mg/L F^−^. Therefore, these 3 *CsXTH* genes might be able to respond to the stress of high concentrations of F^−^. The expression patterns of *CsXTHs* in the same subclass were also similar under F^−^ treatments. For example, 75.0% of *CsXTHs* in subclass I and 66.7% of *CsXTH* genes in subclass IIIB all had the highest expression levels under 8 mg/L F^−^ treatments.

Previous studies showed that cell walls had highly complex and dynamic structures composed of cellulose, hemicellulose, pectic polysaccharides and structural proteins, which had strong abilities of accumulating cations [48]. The main components such as pectin and hemicellulose could quickly combine with Al^3+^, resulting in the accumulation of more than 80–90% Al in the cell walls. Therefore, the elasticity and water conductivity of cell walls significantly decreased, and the elongation of roots would be inhibited [4]. The content of xyloglucan in different substitution degrees in roots of *C. sinensis* was determined by immunofluorescence labeling (Fig. 9). There was more xyloglucan in root tips when the *C. sinensis* were deficient in Al. With the increase of Al^3+^ concentration, the content of xyloglucan oligosaccharide in roots increased and the content of xyloglucan decreased. When the Al^3+^ concentration reached 0.4 mM, the content of xyloglucan oligosaccharide in roots was the highest. Studies reported that appropriate concentration of Al^3+^ could not only promote the development of roots, but also increase the chlorophyll content in tea leaves and enhance the net photosynthesis of *C. sinensis* [49]. The formation of xyloglucan oligosaccharide in roots was gradually inhibited with the further increase of Al^3+^ concentration. And this inhibition was more obvious at 4.0 mM Al^3+^, which would not conducive to the cell wall reconstruction and growth of roots [50]. Luo et al. (2006) reported that there was a very serious inhibition on the growth of *C. sinensis* when the Al^3+^ concentration was greater than 1.85 mM [45]. The treatment of 8 mg/L F^−^ promoted the content of xyloglucan oligosaccharide. When the F^−^ concentration reached 16 mg/L, the content of xyloglucan oligosaccharide decreased, which was adverse to cell wall reconstruction and root growth. Although for some plants, the growth and development might be inhibited only when the F^−^ concentration reached 30 mg/L, which was supposed to be caused by species differences [51].

Previous studies showed that there was a good correlation between the activity of XET and the growth rate and the elongation of cell wall [15, 52–54]. Vissenberg et al. (2005) reported that the activity of XET was high near the starting point of root hair, and it was believed that XET activity was all high in elongation region of roots in various vascular plants [55]. According to the determination results of XET activity in roots, when the Al^3+^ concentration was 0.4 mM or the F^−^ concentration was 8 mg/L, the XET activity was significantly higher than that of the control group (Fig. 10). However, the activity also decreased if the concentrations of Al^3+^ or F^−^ continued to rise, which was consistent with the results of immunofluorescence labeling. It was indicated that 0.4 mM Al^3+^ or 8 mg/L F^−^ could promote the activity of XTH, which might be was beneficial to the cell wall reconstruction and elongation of roots. Based on the results of immunofluorescence labeling and XET activity, it was probable that CsXTHs could get involved in catalyzing the synthesis of xyloglucan oligosaccharide from single xyloglucan molecules, which could loosen cell walls and stimulate the initiation of root hair. However, the direct effect and evidence verifying the functions of CsXTHs still remained to be found and studied.

In summary, the bioinformatics characteristics, expression patterns and the activities of *XTH* genes in *C. sinensis* under Al^3+^ or F^−^ treatments were explored. Under a low concentration of Al^3+^ or F^−^ (0.4 mM Al^3+^ or 8 mg/L F^−^), the expression levels of *CsXTHs*, the content of xyloglucan oligosaccharide and the activity of XET were promoted. According to these results, we supposed 0.4 mM Al^3+^ or 8 mg/L F^−^ could stimulate XTH activity by regulating the expressions of *CsXTH* genes, which might further participate in the transformation of xyloglucan, affect the cell wall reconstruction and ultimately induce root elongation and growth of *C. sinensis*.

## Materials and methods

### Identification and characteristics of *XTH* gene family in *C. sinensis*

A total of 45 sequences with annotations related to *XTH* genes were searched from the *C. sinensis* transcriptome database (PRJNA315669) [22]. Then, 20 candidate genes of *XTH* were screened from 45 sequences using the BLAST program of NCBI database online search tool (https://blast.ncbi.nlm.nih.gov/Blast.cgi). All candidate genes with complete domains were analyzed using multi-sequence alignment in ClustalX l.83 software program (Thompson et al., 1997) to eliminate repeated sequences. Finally, 14 *CsXTH* genes were obtained for this study and the coding sequences (CDS) of *CsXTHs* were shown in Additional file 1.

The physicochemical characteristics of 14 CsXTH proteins were obtained from the online tool of ExPASy-ProtParam (https://web.expasy.org/protparam/). The gene structures were constructed using the online software of GSDS 2.0 (https://gsds.cbi.pku.edu.cn/).

### Phylogenetic tree construction, conserved motif analysis and sequence alignment

A total of 33 XTH protein sequences of *A. thaliana* were downloaded from The Arabidopsis Information Resource (TAIR) (http://www.arabidopsis.org). A neighbor-joining (NJ) phylogenetic tree with a bootstrap value of 1000 was constructed by using MEGA version 6.0 software (Tamura et al., 2011).

The online software MEME (http://meme-suite.org/tools/meme) was used to analyze the conserved motifs with the parameters of 20 motifs and E < 1e^− 10^. And the conserved motifs were visualized with TBtools software (https://github.com/CJ-Chen/TBtools). In addition, DNAMAN software (Lynnon Biosoft) was used to multiple alignments of CsXTH proteins.

### Plant materials and treatments

Annual seedlings of *C. sinensis* cv. Longjing 43 (Nanjing Ya Run Tea Co., Ltd., China) with consistent growth were selected, cleaned and cultivated under hydroponic culture, with a light cycle of 12 h/12 h (light/dark), a light intensity of 4000 lx, relative humidity of 70% and temperatures of 25 °C/23 °C (day/night). The nutrient solutions were prepared according to the method of Wan et al. (2012) and were changed once a week [56]. After the length of new roots exceeded 5 cm, these seedlings were treated respectively with different concentrations of Al^3+^ and F^−^. The concentrations of Al^3+^ (AlCl_3_) treatments included 0 mM, 0.1 mM, 0.4 mM, 2.0 mM and 4.0 mM. And the concentrations of F^−^ (NaF) treatments contained 0 mg/L, 8 mg/L and 16 mg/L. The new roots were collected after 24 h of treatments, washed with deionized water and stored at − 80 °C for total RNA extraction. In order to detect the expression profiles of *XTH* genes in different tissues, roots, stems, old leaves, young leaves, flowers, pollen and fruits were also collected from seedlings of *C. sinensis* under normal growth conditions with 3 biological replicates and stored at − 80 °C for total RNA extraction.

### Immunofluorescence detection of xyloglucan

To test the responses of cell walls under different concentrations of Al^3+^ and F^−^ treatment, we used immunostaining-monoclonal antibodies (LM10 and LM25) (Paul Knox Cell Wall Lab of Leeds University, UK) that could substitute specific degrees of xyloglucan as previously reported [30]. LM10 and LM25 were specific immunostaining sites for xyloglucan. LM10 could indicate xyloglucan, while LM25 could indicate xyloglucan oligosaccharide (mainly XXLG, XLLG, XXLG and XXXG). The fluorescence intensity of LM10 or LM25 represented the content of xyloglucan or xyloglucan oligosaccharide.

The new roots of seedlings were collected and washed with deionized water after 24 h of treatments of Al^3+^ (0, 0.1, 0.4, 2.0, 4.0 mM) and F^−^ (0, 8, 16 mg/L). The transverse sections were sliced at 0–2 mm from the root tip. The thickness of each section was less than 300 μm. More than 15 sections were sliced of each treatment group with 3 biological replicates. The sections were fixed into 4% paraformaldehyde solution at 25 °C for 2 h and then washed with PBS (phosphate buffer solution) (pH 7.4). The sections were transferred to PBS containing 0.2% BSA (bovine serum albumin) and blocked for 0.5 h. Then the sections were washed again with PBS to remove buffer residue. After that these sections were placed in primary antibody (LM10 and LM25) diluted 10 times at 37 °C for 2 h and then washed with PBS. FITC (fluorescein isothiocyanate) (Solarbio Science&Technology, China) was diluted for 50 times for incubation as secondary antibody. The sections were incubated at 37 °C for 2 h and then washed with PBS. Finally, the sections were observed and photographed with an Ultra high resolution confocal microscope (Zeiss LSM800, Germany).

### Determination of XET activity

In order to investigate the effect of different concentrations of Al^3+^ and F^−^ on the activity of XET, we used the double antibody sandwich method to detect the activity of XET [52].

### RNA extraction and qRT-PCR analysis

Total RNA was extracted from samples by using EASYspin RNA Extraction Kit (Adelaide Biological, China), according to the manufacturer’s instructions. Total RNA isolated from samples was reverse transcript into cDNA using the PrimeScript™ RT reagent Kit with gDNA Eraser (Perfect Real Time) kit (Takara Biomedical Technology, China). The expression levels of *CsXTH* genes were detected on a Quantitative real-time PCR system (Bio-rad CFX96, USA) by using the SYBR Premix Ex Taq kit (Takara Biomedical Technology, China). Each reaction contained 7.2 μL ddH_2_O, 10 μL 2 × SYBR Premix, 2 μL diluted cDNA and 0.4 μL gene specific primers. The qRT-PCR conditions were as follows: 95 °C for 30 s, 40 cycles at 95 °C for 5 s, 60 °C for 30 s and 95 °C for 10 s. Second, 65 °C for 5 s and 95 °C for 5 s. Experiments were repeated with 3 independent biological replicates, and the relative expression levels were analysed by the 2^-ΔΔCT^ method. Specific primers for 14 *CsXTH* genes were designed using Primer Premier 5 software (PREMER Biosoft International), and *Csβ-actin* was used as an internal reference gene (Table 2).

**Table 2:** Primers used for qRT-PCR

Primer	Forward primer sequence	Reverse primer sequence
*β-actin*	CTCAGTCCAAAAGAGGTATTCT	GTAGAATGTGTGATGCCAGATC
*CsXTH1*	AAGTGGTTTCAAGTCCCTG	CTCTATGTCAATCTCATCGTG
*CsXTH2*	GATTGGGCAACAAGAGGTG	GCTTTGAGTCCGTGCAGTAA
*CsXTH3*	GACCACCTTCACCTGGCTAC	TGGATGTGCTTCGTTGTTTG
*CsXTH4*	ACCCTACTACCGATTTCCA	CAGTCTTCACAAGTCCACCT
*CsXTH5*	TGTCAAGGCAAGGGTAATAGA	GTAGCCCAATCATCAGCGT
*CsXTH6*	ATGATACAGGTTGTGGGTTT	ATCTCACGGTTTCCAGTTC
*CsXTH7*	GGAACAGAACAGGGCAACC	GGAAACCTCACTCCCAAATC
*CsXTH8*	TTGTTATGACACGGTAAGGTA	ATGCTGGTCTCCATAGTTCTT
*CsXTH9*	GGTCCTCAGCATCAAAGA	AATCCTCCCATCCCCAC
*CsXTH10*	TGCTTCTATCAAACTTCCTGC	CCCTCCCATTGCTTCTGT
*CsXTH11*	CCACCAAGGACTTCCACTC	AATCGTCTGCGTTCCAAAT
*CsXTH12*	TATGTTGATGATGTCCCAATAAGAG	AGACGAAGGGCTGGAAGCGGTAGTT
*CsXTH13*	ACGCTGATGACTGGGCTAC	TTTGAACAGGAAGAGGAGGAA
*CsXTH14*	GGTCAAAGGGTCCATTCATAG	TCTCATTCTGCCACCAGTTAGT

### Statistical analysis

The experimental data were analyzed using IBM SPSS statistics 20 and Excel 2007. The difference of results under different concentrations of Al^3+^ and F^−^ treatment was determined by the multiple of Duncan (*p* < 0.05).

## Supplementary Information

**Additional file 1: **The CDS of *CsXTHs*. The coding sequences (CDS) of 14 *XTH* genes from *Camellia sinensis*.

## Data Availability

The *C. sinensis* transcriptome datasets analyzed during the current study are available in the National Center for Biotechnology Information (https://www.ncbi.nlm.nih.gov) under the accession number: PRJNA315669. The plant samples used in this study were deposited at Laboratory of Tea breeding and cultivation, Nanjing Agricultural University, Nanjing, China.

## Abbreviations

XTH: Xyloglucan endotransglycosylase/hydrolases
XET: Xyloglucan endotransglucosylase
XEH: Xyloglucan endohydrolase
Cys: L(+)-Cysteine
